# Minocycline attenuates post‐operative cognitive impairment in aged mice by inhibiting microglia activation

**DOI:** 10.1111/jcmm.12854

**Published:** 2016-04-06

**Authors:** Hui‐Lin Wang, Hua Liu, Zhang‐Gang Xue, Qing‐Wu Liao, Hao Fang

**Affiliations:** ^1^ Department of AnesthesiologyZhongshan HospitalFudan UniversityShanghaiChina; ^2^ Department of AnesthesiologyShanghai Ninth People's HospitalShanghai Jiao Tong University School of MedicineShanghaiChina; ^3^ Department of AnesthesiologyJinshan HospitalFudan UniversityShanghaiChina

**Keywords:** POCD, minocycline, microglia, isoflurane

## Abstract

Although it is known that isoflurane exposure or surgery leads to post‐operative cognitive dysfunction in aged rodents, there are few clinical interventions and treatments available to prevent this disorder. Minocycline (MINO) produces neuroprotection from several neurodegenerative diseases and various experimental animal models. Therefore, we set out to investigate the effects of MINO pre‐treatment on isoflurane or surgery induced cognitive impairment in aged mice by assessing the hippocampal‐dependent spatial memory performance using the Morris water maze task. Hippocampal tissues were isolated from mice and evaluated by Western blot analysis, immunofluorescence procedures and protein array system. Our results elucidate that MINO down‐regulated the isoflurane‐induced and surgery‐induced enhancement in the protein levels of pro‐inflammatory cytokine tumour necrosis factor alpha, interleukin (IL)‐1β, interferon‐γ and microglia marker Iba‐1, and up‐regulated protein levels of the anti‐inflammatory cytokine IL‐4 and IL‐10. These findings suggest that pre‐treatment with MINO attenuated isoflurane or surgery induced cognitive impairment by inhibiting the overactivation of microglia in aged mice.

## Introduction

Post‐operative cognitive dysfunction (POCD), a major clinical issue, is an impairment of recent memory, concentration, language comprehension and social integration. It is reported that at the time of discharge from the hospital, 41.4% of elderly patients (60 years or older) were subjected to POCD after non‐cardiac surgery 1. Post‐operative cognitive dysfunction diminishes the quality of the patient's life and adds cost to hospitalization and out‐of‐hospital care. Post‐operative cognitive dysfunction has also been associated with an increase in surgical morbidity and mortality. Its avoidance and treatment therefore represents one of the greatest challenges for the perioperative physician dealing with an elderly surgical population 2, 3.

Many pathophysiological mechanisms have been implicated in development of POCD, but the exact cascade leading to its development remains elusive. Recent animal studies have strongly suggested the role of neuroinflammation in the development of neurodegenerative diseases and the occurrence of cognitive deficits associated with ageing 4, 5, 6, 7, 8. Neurodegenerative diseases such as Parkinson's and Alzheimer's disease, mild cognitive impairment and POCD could be viewed as a disease continuum sharing similar pathophysiologic mechanisms 9, 10. In the past decade, inflammation has emerged as a key event in the progression of Alzheimer's disease and Parkinson's disease 11, 12. Age‐related neuroinflammatory changes have also been reported, including the increased expression of pro‐inflammatory cytokines interleukin (IL)‐1β, IL‐6 and tumour necrosis factor alpha (TNF‐α) 13. The age‐related change in the inflammatory profile of the brain likely results from alterations in the activation status of the brain's primary immune cell, microglia. Research has established that microglia from aged animals are primed to express an inflammatory phenotype 14, 15. In the ageing brain, microglia respond to stimulus by producing more pro‐inflammatory cytokines (*e.g*. IL‐1β) and producing them for longer than microglia in younger brains. Additionally, study found that 50% of elderly patients with mild cognitive impairment had increased microglia activation compared to age‐matched controls 8. These data provide evidence that normal ageing increases immune activation within the brain, but additional work is needed to confirm this link and determine the role microglial cells play in maintenance of these deficits.

Isoflurane may increase the levels of pro‐inflammatory cytokines, which may cause neuroinflammation 16. Isoflurane impaired the long‐term spatial reference memory and hippocampus‐dependent learning and memory by activating the IL‐1β. Interleukin‐1β increased activated caspase 3 in the hippocampus and decreased the neuronal density in the CA1 region. This activation ultimately led to neurodegeneration and cell death in the hippocampus 17, 18.

Hippocampal neuroprotection has been proved to improve spatial learning and memory following cerebral ischaemia 19. Minocycline (MINO), a tetracycline derivative, has been reported to have neuroprotective effects caused by the inhibition of inflammation and microglial activation 20, 21, 22. Therefore, the present study tested the hypothesis that pre‐treatment with MINO would improve spatial leaning memory in aged mice by inhibiting microglial cell activation and reducing release of hippocampal pro‐inflammatory cytokines.

## Materials and methods

### Animal experiments

Aged mice weighing 35–40 g were divided, respectively, into six groups: control (Con), MINO, isoflurane (Iso), minocycline‐isoflurane (MINO‐Iso), surgery (Sur) and minocycline‐surgery (MINO‐Sur) (*n* = 20 in each group). Mice in the Mino, Mino‐Iso and Mino‐Sur groups received MINO (45 mg/kg) (Wyeth Pharmaceutical Co. Ltd, Suzhou, China) by intraperitoneal injection 12 hrs before exposure to isoflurane or appendectomy. The MINO dose used in our study in reference to other studies 1, 23, 24. Mice in the Iso or Sur group were subjected to water maze test at day 3 and day 28 and harvested their hippocampi at day 3, day 14 and day 28 for measuring inflammatory cytokines [IL‐1β, TNF‐α, interferon (IFN)‐γ, IL‐4 and IL‐10] and microglia marker Iba‐1. All C57BL/6 mice were purchased for Shanghai Laboratory Animal Center of the Chinese Academy of Sciences and maintained in pathogen‐free conditions. All animals used were accordant with the guidelines of the Institutional Animal Care and Use Committee of Fudan University (Shanghai, China).

### Anaesthesia procedure

Mice in the Iso exposure group, that were assigned randomly, received 1.4% isoflurane in 50% oxygen for 2 hrs at a flow rate of approximately 3 l/min. in a Plexiglas anaesthetizing chamber, which was adjusted to maintain constant levels of minimum alveolar concentration, oxygen and carbon dioxide. Gases within the anaesthetic chamber were monitored continuously, and during anaesthesia, a pulse oximeter was used to measure arterial oxygen saturation non‐invasively. Mice were breathing spontaneously, and the temperature of the anaesthetizing chamber was controlled by using a heating pad to maintain the temperature of the mice at 37 ± 0.5°C. At the end of the anaesthesia procedure, anaesthetics was discontinued. Mice were then recovered for 20 min. in a chamber gassed with 50% O_2_ and at 37°C and then place them in their home cage. To avoid the confounding influence of residual anaesthetic, mice were allowed to recover for 48 hrs.

### Surgical procedure

Mice in the Sur and Mino‐Sur groups, that were assigned, randomly, were anaesthetized with pentobarbital sodium under sterile conditions. All surgical interventions on the mice were taken by a standardized procedure, a midline laparotomy was taken firstly, then the appendix was mobilized and exteriorized. Division of the appendix was performed between two ligatures that were placed proximal to the border of the caecum and appendix. The caecal stump was irrigated with saline. In the end, the two layers of the abdominal wall were closed by a running suture technique. Mice were breathing spontaneously, and a heating pad was used to maintain the temperature at 37 ± 0.5°C. In the recovery period of 2 days after surgery, mice were assessed daily.

### Morris water maze

Morris water maze task was used to assess hippocampal‐dependent spatial memory performance. The maze consisted of a circular tub (100‐cm diameter) and a clear mesh plastic square platform (8.5 cm). The platform was submerged 1 cm under the surface of the water. To conceal the platform white tempera paint was used to make the water opaque. During the test, the water temperature was maintained at 21 ± 1°C. Extra‐maze cues were located around the maze. Four trials (up to 60 sec.) were taken on every mice per day from different start locations for five consecutive days. Mice that found the platform were allowed to stay on it for 15 sec. If a mouse did not find the platform within a 60‐sec. period, it was gently guided to the platform and allowed to stay on it for 15 sec. The swimming motions of the animals were recorded by a video tracking system, and the data were analysed using motion‐detection software for the MWM (Institute of Materia Medica, Chinese Academy of Medical Sciences & Peking Union Medical College, Beijing, China). A single probe trial was conducted on day 6. During the testing trials, the platform was removed and the mouse was placed in the opposite quadrant. Each mouse was allowed to swim for 60 sec., and the number of times the animal crossed the original location of the platform was recorded by the tracking system. Specifically, before being returned to its regular cage, each mouse was placed in a holding cage under a heat lamp for 1–2 min. to dry after every trial.

### Western blot and antibody

Hippocampal tissues were isolated from the mice in the Iso or Sur group and homogenated in RIPA (Radio Immunoprecipitation Assay) buffer (Cell Signaling, Boston, USA) plus protease inhibitors on ice for 30 min. and then centrifuged at 12000 r.p.m. at 4°C for 15 min. The supernatant was recovered and the protein concentration determined by BCA (Bicinchoninic acid) method (Bio‐Rad, Hercules, CA, USA). Identical amounts of protein were subjected to SDS‐PAGE (12% gels) and then blotted onto pre‐activated polyvinylidene difluoride membrane. Membranes were incubated with 5% fat‐free milk for 2 hrs at room temperature and incubated with primary antibodies at 4°C overnight, blocked with secondary antibodies for 1.5 hrs at room temperature. Finally, the bands were visualized with an ECL detection kit (Pierce, IL, USA). The primary antibodies used for immunoblotting were a mouse monoclonal anti‐GAPDH antibody (1:2000; A2228;Sigma‐Aldrich, St. Louis, MO, USA) and anti‐Iba‐1 antibody (1:1000;Ab107159; Abcam, Cambridge, MA, USA).

### Immunofluorescence procedures

Firstly, the hippocampal tissue sections were prepared. The mice were sacrificed in different groups and transcardially perfused with ice‐cold PBS and 4% paraformaldehyde. The brains were then dissected and the meninges were removed carefully and then fixed with the same fixative overnight, cryoprotected by first sinking in 10% and then in 30% sucrose (in 0.1 M phosphate buffer) at 4°C. The tissue sections were blocked with PBS with 0.5% Tween‐20 (PBST) containing 10% donkey serum and 0.5% Triton‐100 for 1 hr at room temperature. After overnight incubation at 4°C with the indicated primary antibody: Iba‐1 antibody (ab107159, 1:200; Abcam), cells were washed with PBS (3× 10 min.) and then incubated for 2 hrs at room temperature with a 1:2000 dilution of anti‐goat IgG secondary antibody (Invitrogen, Carlsbad, CA, USA). Tissue sections were exposed for 5 min. to 0.5 μg/ml DAPI (Sigma‐Aldrich) at room temperature, coverslips were mounted using Fluoromount Aqueous Mounting Medium (Sigma‐Aldrich).

### Quantification of IL‐1β, TNF‐α, IFN‐γ, IL‐4 and IL‐10 by Bio‐Plex protein array system

Hippocampal tissues of mice in the Iso or Sur group were lysed on ice in 20 mM Tris–HCl buffer (pH = 7.3) containing protease inhibitors (Roche Diagnostics, Basel, Switzerland), then the homogenates were centrifuged at 15,000 × g for 15 min. at 4°C. The supernatant was ultracentrifuged at 150,000 × g for 2 hrs 4°C. Protein concentrations in the supernatants were determined using the Bradford protein assay. IL‐1β, TNF‐α, IFN‐γ, IL‐4 and IL‐10 were measured in the hippocampus as previously described 25. In brief, a customized 5‐plex mouse cytokine panel consisting of fluorescent beads for IL‐1β, TNF‐α, IFN‐γ, IL‐4 and IL‐10 (Bio‐Rad) was analysed with a Luminex protein suspension array system (Bioplex 200; Bio‐Rad) according to manufacturer's instructions. As a result of the large binding surface of the beads, this assay is highly sensitive and has been proven before to work well for detecting cytokines from brain tissues 26. All samples were run in triplicate and data were analysed with the Bio‐Plex Manager software. The results were expressed as pg/ml of brain supernatant.

### Statistical analysis

All data were presented as mean ± S.E.M. Statistical Package for the Social Sciences (SPSS) v.19.0 software (IBM, Chicago, IL, USA) was used for statistical analyses. Behavioural studies were analysed using two‐way anova with repeated measures. Other data were analysed with one‐way anova, followed by a least square difference multiple comparison test. A *P* value of <0.05 was considered statistically significant.

## Results

### Minocycline pre‐treatment improved cognitive function after isoflurane exposure or surgery

**Figure 1:**
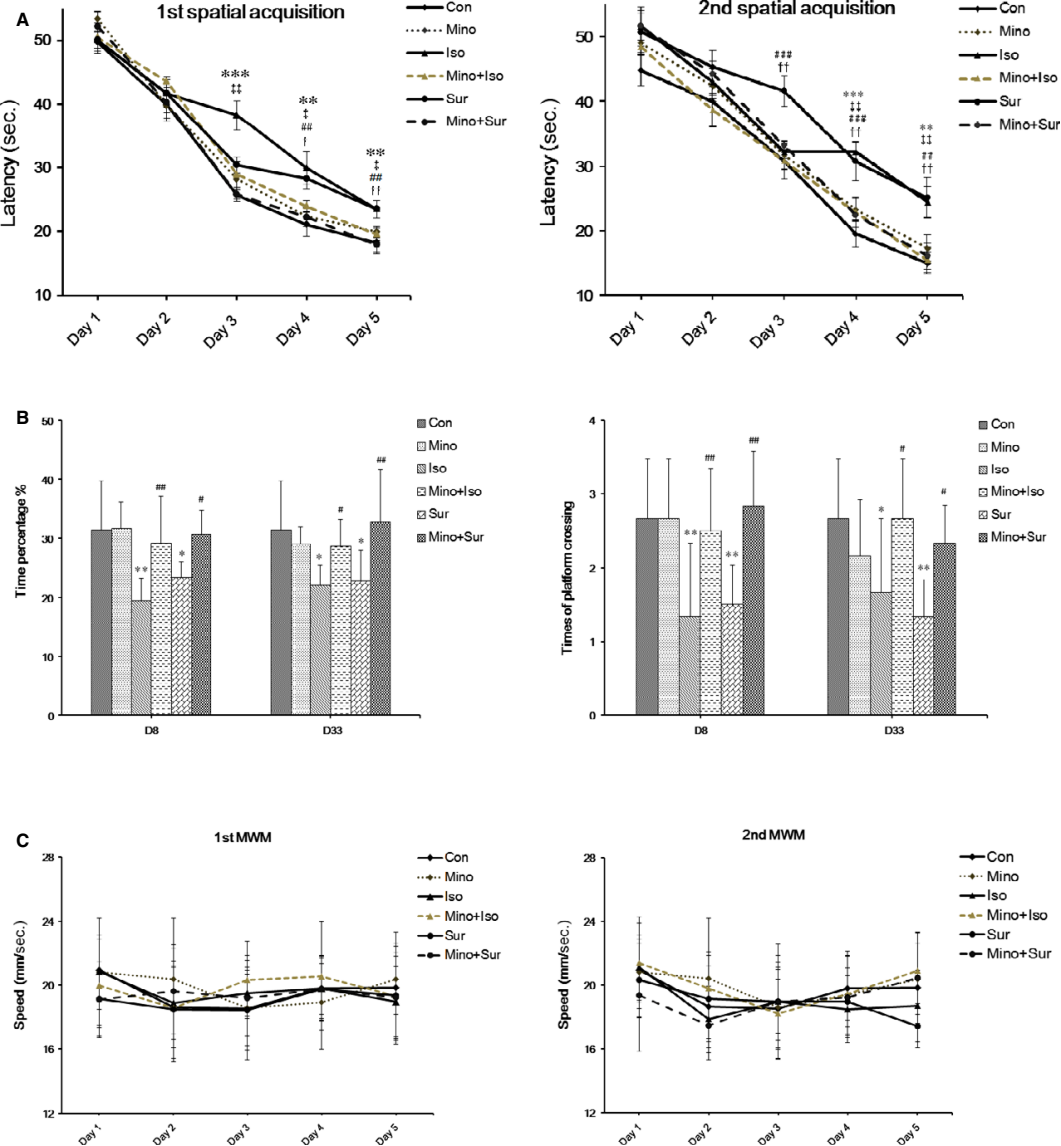
Minocycline pre‐treatment prevented spatial memory impairment induced by isoflurane exposure or appendectomy. (**A**) The mean escape latency (in seconds) to find the hidden platform across training days. In the Iso group, the significant differences were found on day 3, day 4 and day 5 at the first spatial acquisition and day 4 and day 5 at the second spatial acquisition. In the Sur group, the significant differences were found on day 4 and day 5 at the first spatial acquisition and day 3, day 4 and day 5 at the second spatial acquisition. Treatment with minocycline shortened the mean escape latency (**P < 0.01, ***P < 0.001 Iso *versus* Con; ##*P* < 0.01, ###*P* < 0.001 Sur *versus* Con; ‡*P* < 0.05, ‡‡*P* < 0.01 Mino+Iso *versus* Iso, †*P* < 0.05, ††*P* < 0.01 Mino+Sur *versus* Sur). (**B**) Probe trial performance of during testing. Both Iso and Sur group spent less time in the target quadrant searching for the missing platform and less number of times of crossing the former location of the platform than did the Con, Mino+Iso and Mino+Sur group at post‐anaesthesia/post‐surgery day 8 and 33. (**C**) There were no differences in the swimming speed among all groups during training days. *N* = 12 for each group.

As shown in Figure 1A, mice in Iso and Sur groups showed a decrease in latency. In the place trial, the mice in the Mino+Iso/Mino+Sur group spent less time to find the platform than those in the Iso/Sur group. In the probe trial (Fig. 1B), both Iso and Sur group spent less time in the target quadrant searching for the missing platform and less number of times of crossing the former location of the platform than did the Con, Mino+Iso and Mino+Sur group at post‐anaesthesia/post‐surgery day 8 and 33. Swimming speeds were also analysed during place trials, and no differences were observed among the six groups (Fig. 1C).

### Minocycline reduced the isoflurane‐induced or surgery‐induced up‐regulation of pro‐inflammatory cytokine levels in the hippocampus

**Figure 2:**
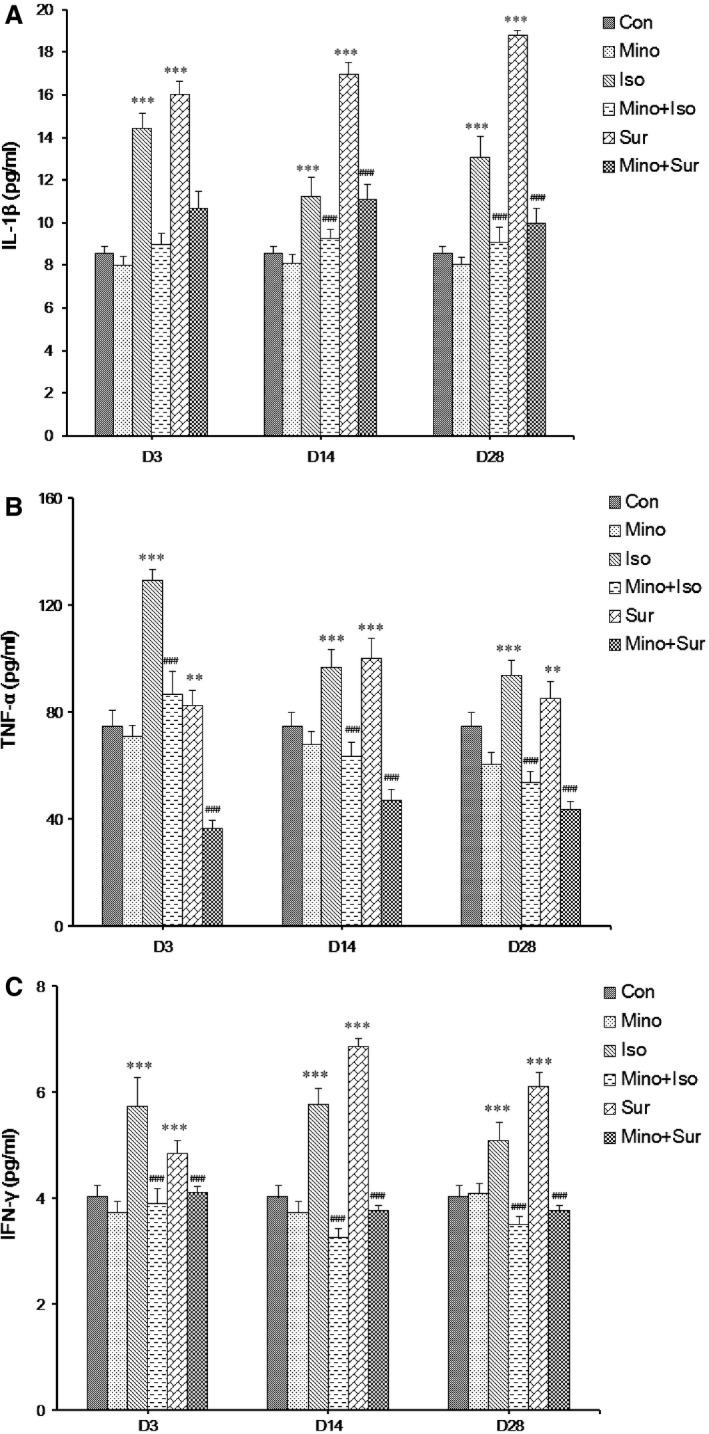
The effect of minocycline on the expression of pro‐inflammatory cytokine IL‐1β, TNF‐α and IFN‐γ in hippocampus. (**A**) Pre‐treatment with minocycline led to a decrease in the levels of IL‐1β protein that were up‐regulated by isoflurane exposure or appendectomy. (**B**) Pre‐treatment with minocycline led to a decrease in the levels of TNF‐α protein that were up‐regulated by isoflurane exposure or appendectomy. (**C**) Pre‐treatment with minocycline led to a decrease in the levels of IFN‐γ protein that were up‐regulated by isoflurane exposure or appendectomy. The results are presented as the mean ± S.E.M. (*n* = 6) (**P < 0.01, ***P < 0.001 Iso *versus* Con, Sur *versus* Con; ###*P* < 0.001 Mino+Iso *versus* Iso, Mino+Sur *versus* Sur).

The levels of pro‐inflammatory cytokine IL‐1β, TNF‐α and IFN‐γ detected at a series of time‐points after anaesthesia or surgery were respectively shown in Figure 2A–C. After isoflurane exposure or appendectomy, the expression of IL‐1β, TNF‐α and IFN‐γ increased significantly. The level of IL‐1β peaked 3 days after isoflurane exposure and 28 days after appendectomy (Fig. 2A); the level of TNF‐α and IFN‐γ peaked 3 days after isoflurane exposure and 14 days after appendectomy (Fig. 2B and C). When mice were pre‐treated with MINO, the elevated levels of IL‐1β, TNF‐α and IFN‐γ in the hippocampus were reversed.

### Minocycline decreased the protein level of Iba‐1 in the hippocampus

**Figure 3:**
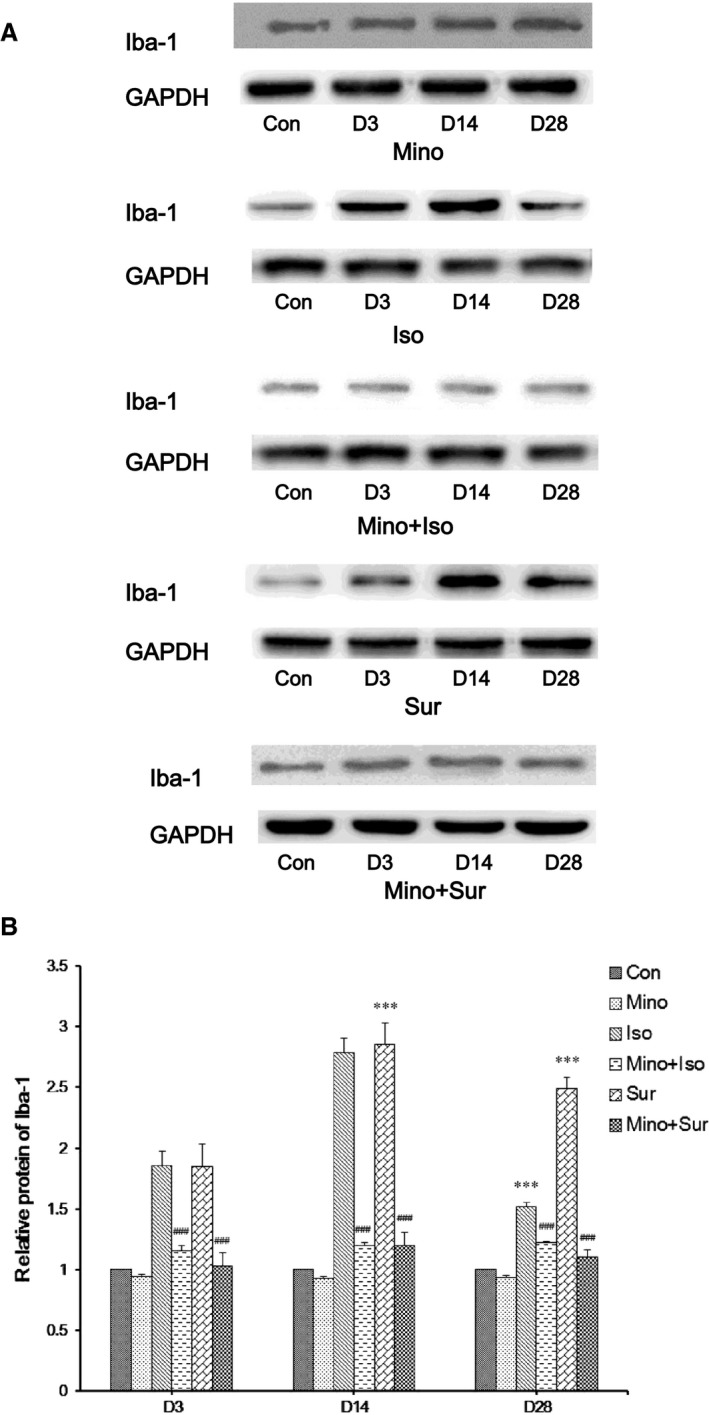
The effect of minocycline on the expression of Iba‐1 in hippocampus. (**A**) Western blot analysis of Iba‐1 protein levels. (**B**) Quantification of relative protein results showed that minocycline decreased the expression of Iba‐1 after isoflurane exposure or appendectomy in aged mice. The results are presented as the mean ± S.E.M. (*n* = 4) (***P < 0.001 Iso *versus* Con, Sur *versus* Con; ###*P* < 0.001 Mino+Iso *versus* Iso, Mino+Sur *versus* Sur).

To investigate the potential mechanism of how inhaled anaesthetics or surgical trauma sensitizes microglial activation, Iba‐1 protein level was detected by Western blot (Fig. 3A). As shown in Figure 3B, isoflurane exposure and appendectomy markedly increased the expression of Iba‐1 in the hippocampus, which was reversed by treatment with MINO.

### Minocycline decreased the Iba‐1‐positive cell number in the hippocampus

**Figure 4:**
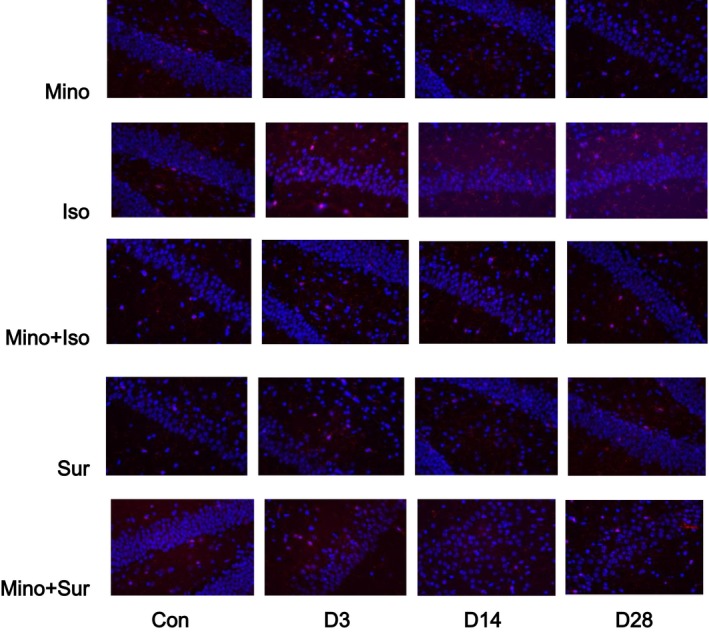
Immunofluorescent staining of hippocampal Iba‐1. Isoflurane exposure and appendectomy exhibited an increase number in Iba‐1‐positive cells in hippocampus D3, D14 and D28, which was reversed by treatment with minocycline.

To investigate the potential mechanism of how inhaled anaesthetics or surgical trauma sensitizes microglial activation, immunofluorescent staining of hippocampal Iba‐1 in the six groups were performed. As shown in Figure 4, isoflurane exposure and appendectomy exhibited an increase number in Iba‐1‐positive cells in hippocampus D3, D14 and D28. Pre‐treatment with MINO led to a decrease number in Iba‐1‐positive cells that were up‐regulated by isoflurane exposure or appendectomy.

### Minocycline increased anti‐inflammatory cytokine levels in the hippocampus after isoflurane or surgery

**Figure 5:**
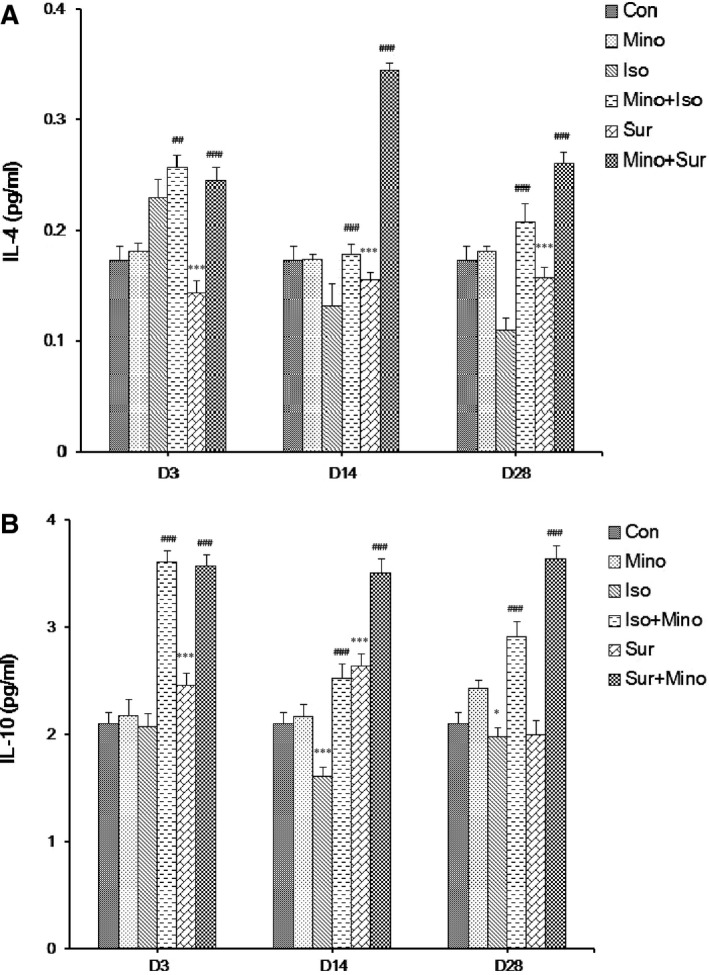
The effect of minocycline on the expression of anti‐inflammatory cytokine IL‐4 and IL‐10 in hippocampus. (**A**) IL‐4 expression increased 3 days and then decreased 14 and 28 days after isoflurane exposure; it decreased significantly after appendectomy. Pre‐treatment with minocycline led to an increase in the levels of IL‐4 protein. (**B**) IL‐10 expression decreased at 14 and 28 days after isoflurane exposure but increased at 3 and 14 days after appendectomy. Pre‐treatment with minocycline led to an increase in the levels of IL‐10 protein. The results are presented as the mean ± S.E.M. (*n* = 6) (*P < 0.05, **P < 0.01, ***P < 0.001 Iso *versus* Con, Sur *versus* Con; ##*P* < 0.01, ###*P* < 0.001 Mino+Iso *versus* Iso, Mino+Sur *versus* Sur).

We also detected anti‐inflammatory cytokine IL‐4 and IL‐10 protein levels at a series of time‐points after anaesthesia or surgery, which were respectively shown in Figure 5A and B. Interleukin‐4 expression increased 3 days and then decreased 14 and 28 days after isoflurane exposure; it decreased significantly after appendectomy. When pre‐treated with MINO, the level of IL‐4 increased (Fig. 5A). Interleukin‐10 expression decreased at 14 and 28 days after isoflurane exposure but increased at 3 and 14 days after appendectomy. When pre‐treated with MINO, the level of IL‐10 increased significantly (Fig. 5B).

## Discussion

The neuropathogenesis of POCD remains largely to be determined, there are few clinical interventions and treatments available to prevent this disorder 27, 28. Our recent research show that the pathway of microglia activation and pro‐inflammatory cytokine release contributed to the neurotoxicity and impairment of learning and memory induced by isoflurane or surgery 29. Minocycline can inhibit the activation of microglia function. We, therefore, investigate the effect of MINO on isoflurane or surgery induced cognitive disorder in aged mice. The major finding of this study was that the latency of group Isoflurance anaesthesia and appendectomy have declined as a result of pre‐treatment with MINO, whereas the frequency of crossing the former location of the platform and the period of staying on platform has increased significantly. Additionally, MINO treatment suppressed the excessive release of pro‐inflammatory cytokines (IL‐1β, TNF‐α and IFN‐γ) and increased the release of anti‐inflammatory cytokines (IL‐4 and IL‐10) in the hippocampus induced by isoflurane or appendectomy by inhibiting the overactivation of microglia.

To determine the effect of MINO on cognitive function after isoflurane anaesthesia or appendectomy, the Morris Water Maze was used to assess learning and memory in aged mice, which are important aspects of cognitive function. Our results showed that aged mice existed deficits in the hippocampus‐dependent learning and memory as manifested by the longer escape latency to reach the platform, the less time spent in the target quadrant and the fewer times of original platform crossing in the Morris water maze test at day 3 and day 28 after exposure to 1.4% isoflurane for 2 hrs or appendectomy. The deficits could be attenuated by MINO treatment, which was consistent with some other studies 30, 31.

Minocycline, a second‐generation tetracycline derivative, which can cross blood–brain barrier, has been extensively used to restrict pro‐inflammatory cytokine release from microglia 32. In a mouse model of amyelo lateral sclerosis, MINO inhibited differentiation of M1, but not M2, microglia 33. Upon activation, microglia rapidly differentiate into a variety of forms broadly defined as M1 and M2 34, 35. M1 state is associated with mounting a defence against infection and is considered pro‐inflammatory, releasing cytokines including IL‐1β, IL‐6 and TNF‐α 36, 37. These cytokines are essential for the induction and maintenance of the behavioural symptoms of sickness 38 and promote the release of secondary inflammatory mediators including prostaglandins and nitric oxide 39, 40, 41. M1 state can convert to a M2 neuroprotective state, which release anti‐inflammatory cytokines, leading to suppress inflammation, conduct repairs and restore homeostasis 34, 36, 42.

Our results show that the pre‐treatment of MINO improved aged mice spatial memory ability by inhibiting the microglia overactivation and decreasing the release of inflammatory cytokines. Although we did not specifically investigate the state of microglial activation in the current study, it is verified that the elevation of pro‐inflammatory cytokines (*e.g*. TNF‐α, IL‐1β) in the hippocampus of aged mice indirectly indicated the M1‐like activation of microglia. Reducing the proportion of microglia that are reactive or that ‘get stuck’ in the M1 state after stimulation is a priority for reducing age‐related neuroinflammation that may contribute to cognitive ageing and be a pre‐disposing factor for neurodegenerative disease 43. We chose a relatively high dose by intraperitoneal administration, to get reliable and stable effect. Around 45 mg/kg MINO was used in a previous study to improve the long‐term neuroprotective outcome 44. We found that pre‐treatment with MINO decrease the protein levels of Iba‐1 and pro‐inflammatory cytokines (IL‐1β, TNF‐α and IFN‐γ) while increased two anti‐inflammatory cytokines. Therefore, we presumed that the inhibition of microglia activation by using MINO is possibly because of suppressing the M1‐activation of microglia, which was beneficial for the amelioration of memory deficits, and the signal pathway of MINO is related to decrease the release of pro‐inflammatory cytokines (IL‐1β, TNF‐α and IFN‐γ) and increase the release of anti‐inflammatory cytokines (IL‐4 and IL‐10) to improve spatial memory.

In conclusion, pre‐treatment with MINO attenuated isoflurane or surgery induced cognitive impairment by inhibiting the overactivation of microglia in aged mice. Minocycline may be an effective and practical intervention for POCD prevention.

## Funding

This project was supported by Shanghai Municipal Commission of Health and Family Planning Fund (2013‐314). The funders had no role in study design, data collection and analysis, decision to publish or preparation of the manuscript.

## Conflict of interest

There are no conflicts of interest.

## Author contribution

Conceived and designed the experiments: HF, HLW and ZGX. Performed the experiments: HLW. Analysed the data: QWL and HLW. Wrote the first draft of the manuscript: HLW and HF. Contributed to the writing of the manuscript: HF, HLW and QWL. Made critical revisions and approved final version: HF and ZGX. All authors reviewed and approved of the final manuscript.
